# Cellular and Molecular Mechanisms Explaining the Link Between Inflammatory Bowel Disease and Heart Failure

**DOI:** 10.3390/cells14141124

**Published:** 2025-07-21

**Authors:** Arveen Shokravi, Yuchen Luo, Simon W. Rabkin

**Affiliations:** 1 Department of Medicine, University of British Columbia, Vancouver, BC V6T 1Z4, Canada; 2 Division of Cardiology, University of British Columbia, 9th Floor 2775 Laurel St., Vancouver, BC V5Z 1M9, Canada

**Keywords:** inflammatory bowel disease, heart failure, heart failure with preserved ejection fraction, systemic inflammation, gut microbiome, intestinal barrier dysfunction, dysbiosis, gut–heart axis

## Abstract

Inflammatory bowel disease (IBD), encompassing Crohn’s disease and ulcerative colitis, is increasingly recognized as a systemic condition with cardiovascular implications. Among these, heart failure has emerged as a significant complication. The aim of this narrative review was to explore the cellular and molecular pathways that link IBD and heart failure. Drawing upon findings from epidemiologic studies, experimental models, and clinical research, we examined the pathways through which IBD may promote cardiac dysfunction. Chronic systemic inflammation in IBD, driven by cytokines such as TNF-α and IL-1β, can impair myocardial structure and function. Furthermore, intestinal barrier dysfunction and gut dysbiosis can facilitate the translocation of proinflammatory microbial metabolites, including lipopolysaccharide and phenylacetylglutamine, and deplete cardioprotective metabolites like short-chain fatty acids, thereby exacerbating heart failure risk. Additional contributing factors include endothelial and microvascular dysfunction, autonomic dysregulation, nutritional deficiencies, shared genetic susceptibility, and adverse pharmacologic effects. IBD contributes to heart failure pathogenesis through multifactorial and interrelated mechanisms. Recognizing the role of the gut–heart axis in IBD is crucial for the early identification of cardiovascular risk, providing guidance for integrating care and developing targeted therapies to reduce the risk of heart failure in this vulnerable population.

## 1. Introduction

Inflammatory bowel disease (IBD), primarily composed of Crohn’s disease (CD) and ulcerative colitis (UC), is classically defined as a gastrointestinal disorder but is increasingly recognized as a systemic disease with numerous extraintestinal manifestations affecting numerous organ systems [1,2]. Among these, cardiovascular complications, including myocarditis, pericarditis, arrhythmias, myocardial infarction, and heart failure (HF), have emerged as clinical concerns [3,4].

HF, a clinical syndrome marked by impaired cardiac function, has emerged as an important cardiovascular outcome in the IBD population. Multiple large-scale epidemiological studies have consistently demonstrated this association across diverse populations and healthcare systems. A population-based study in Minnesota found that IBD patients had significantly higher rates of new-onset HF despite a lower prevalence of traditional cardiovascular risk factors [4]. This was further confirmed in a large Swedish cohort study following over 80,000 patients with biopsy-confirmed IBD, which demonstrated a 19% higher risk of developing HF that persisted across all IBD subtypes and patient demographics [5]. Also compelling, a Danish study of over 5 million patients found that the risk of HF was particularly elevated during periods of active IBD flares, with a 2.5-fold increase in the incidence of HF during disease exacerbations [6]. A recent meta-analysis of over 59,000 patients with IBD found consistent evidence of impaired diastolic function, reflected by a reduced E/A ratio, elevated E/e′, and decreased global longitudinal strain [7]. Structural changes were also observed, including increased left atrial size and abnormalities in atrial electromechanical conduction [7].

**Figure 1:**
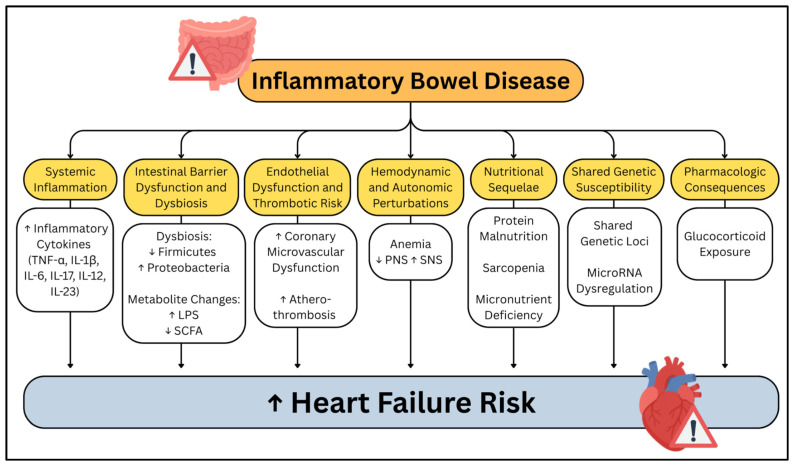
Summary of the mechanisms by which inflammatory bowel disease contributes to the development of heart failure. Yellow boxes represent major inflammatory bowel disease-related pathophysiologic domains, with white boxes provide examples of underlying mechanisms. Arrows indicate directionality, with upward (↑) and downward (↓) arrows denoting increases or decreases in relevant factors.

These epidemiological findings underscore the critical importance of understanding the mechanistic links between IBD and HF. While observational data robustly support this association, the underlying biological pathways remain incompletely characterized. This review examines several key molecular and cellular mechanisms that may link IBD to HF development: systemic inflammation, intestinal barrier dysfunction, gut microbiome alteration, endothelial and vascular abnormalities, thrombotic risk, hemodynamic and autonomic perturbations, metabolic and nutritional consequences, shared genetic susceptibility, and pharmacological influences (Figure 1).

## 2. Methods

Relevant citations were identified through a non-systematic search of PubMed and Google Scholar up to June 2025. Search terms included, but were not limited to, “inflammatory bowel disease,” “heart failure,” “cardiac dysfunction,” and “gut–heart axis”, as well as other specific molecular targets. Artificial intelligence (AI) tools were secondarily used to assist in screening for citations of potential interest; however, any articles identified through AI-assisted search were manually reviewed, and the full original source material was thoroughly evaluated for accuracy and relevance. The reference list of key citations was also screened for additional sources. Priority was given to human studies, meta-analyses, and experimental research directly examining molecular links between IBD and HF, although no formal inclusion or exclusion criteria were applied.

## 3. Systemic Inflammation

### 3.1. Cytokine-Mediated Myocardial Dysfunction

**Figure 2:**
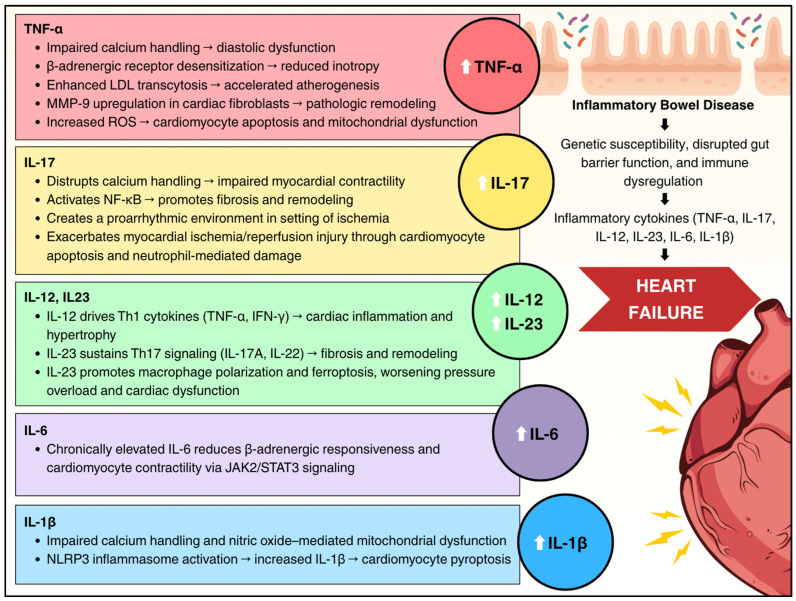
Summary of the effects of elevated cytokines in inflammatory bowel disease on the heart. Colored boxes represent individual cytokines and the associated mechanisms related to heart failure. Upward arrows (↑) denote increased cytokine levels in inflammatory bowel disease; rightward arrows (→) indicate downstream effects. The figure describes the influence of inflammatory bowel disease on subsequent elevation of inflammatory cytokines which drive progression to heart failure.

IBD is characterized by dysregulated cytokine production with prominent elevation of proinflammatory cytokines. These cytokines create a systemic inflammatory milieu that directly impacts cardiac structure and function through multiple interconnected pathways. Figure 2 summarizes the key cytokines that are elevated and involved in IBD and are also implicated in HF, including tumor necrosis factor-alpha (TNF-α), interleukin-1β (IL-1β), interleukin-6 (IL-6), interleukin-12 (IL-12), interleukin-23 (IL-23), and interleukin-17 (IL-17).

### 3.2. Tumor Necrosis Factor-Alpha

TNF-α is an extensively studied cytokine in IBD and cardiac dysfunction and is a common therapeutic target in IBD management. Elevated TNF-α drives adverse left ventricular remodeling through downregulation of sarcoplasmic reticulum Ca^2+^ ATPase gene expression in cardiomyocytes; the impaired calcium reuptake into the sarcoplasmic reticulum during diastole results in diastolic dysfunction [8,9]. TNF-α blunts the inotropic response, and subsequently cardiac contractility, through GRK2-mediated desensitization of β-adrenergic receptors [10]. Additionally, TNF-α amplifies intracellular reactive oxygen species generation, inducing endothelial dysfunction [11], which triggers cardiomyocyte apoptosis [12]. Oxidative stress also contributes to mitochondrial dysfunction, compromising cardiac function [13]. Within the vasculature, TNF-α accelerates atherogenesis by increasing LDL transcytosis and foam cell formation, thereby enlarging atherosclerotic plaques and increasing susceptibility to ischemia [14]. TNF-α also upregulates matrix metalloproteinase-9 in cardiac fibroblasts [15], accelerating extracellular matrix degradation and promoting pathologic ventricular remodeling [16].

### 3.3. Interleukin-1β

IL-1β exerts cardiodepressant effects by impairing calcium handling and inducing nitric oxide (NO)-mediated mitochondrial dysfunction [17]. In IBD, dysregulation of the NLRP3 inflammasome, a multiprotein complex involved in innate immunity, leads to enhanced IL-1β release and induction of pyroptosis, a form of inflammatory programmed cell death [18]. While pyroptosis serves as a defense mechanism by eliminating infected or damaged cells, excessive activation can disrupt the intestinal barrier, promoting further inflammation in IBD [18]. Similarly, in HF, activation of the NLRP3 inflammasome within cardiomyocytes has been shown to induce pyroptosis, contributing to myocardial cell loss and disease progression [19]. Although direct experimental evidence is limited, it is plausible that chronic intestinal inflammation and NLRP3 inflammasome activation in IBD may have systemic effects, promoting cardiac dysfunction.

### 3.4. Interleukin-6

IL-6 levels are also elevated in IBD and HF [20,21]. In IBD, IL-6 is produced by activated immune cells in the intestinal mucosa, promoting chronic inflammation [20]. IL-6 levels are increased in both HF with reduced (HFrEF) and preserved (HFpEF) ejection fraction [22,23]. Chronic elevation in IL-6 depresses β-adrenergic responsiveness and cardiomyocyte contractility, in part through the JAK2/STAT3 pathway [24]. IL-6 binds to its receptor IL-6R, forming a complex that activates the gp130 receptor, which in turn triggers the JAK tyrosine kinase pathway. Activated JAK phosphorylates STAT3, allowing it to dimerize and translocate into the nucleus, where it binds DNA to upregulate genes involved in cardiac inflammation and fibrosis [25].

### 3.5. Interleukin-12 and Interleukin-23

Interleukin-12 (IL-12) and interleukin-23 (IL-23) are proinflammatory cytokines that play an important role in intestinal homeostasis and inflammation in IBD [26,27]. The production of both cytokines is mediated by antigen-presenting cells (APC), including activated dendritic cells and phagocytes [26]. IL-12 promotes intestinal tissue damage through the modulation of Th1 responses, leading to TNF-α and interferon-γ (IFN-γ) secretion, increasing intestinal inflammation [28]. IL-23 is involved in the growth and maintenance of Th17 cells [29]. Together, the IL-23/Th17 axis mediates chronic inflammation through the production of IL-17A, IL-17F, IL-22, IL-26, TNF-a, and IFNγ [26]. IL-12 and IL-23 share a common p40 subunit, which is a therapeutic target for monoclonal antibodies such as ustekinumab [30]. Elevated IL-12 and IL-23 have been previously shown to be associated with cardiovascular diseases [31]. Recent studies have now recognized the role of cardiac inflammation in the pathogenesis of HF and cardiac remodeling through cytokine recruitment and its interplay with the neurohormonal system and endothelial dysfunction [32]. Experimental studies in mice demonstrated that IL-12α KO significantly attenuated pressure overload-induced cardiac inflammation, hypertrophy, fibrosis, and HF progression [33]. Knockout of IL-23p19 in pressure overload mice models has also shown improved cardiac dysfunction and remodeling via attenuation of M1 macrophage polarization and the reduction in ferroptosis [34]. Together, these findings link IL-12- and IL-23-driven inflammation to both intestinal disease and cardiac remodeling, highlighting their role in the pathogenesis of HF.

### 3.6. Interleukin-17

In IBD patients, IL-17A and -17F are significantly upregulated both in inflamed intestinal mucosa and systemically in circulation, with Th17 cells serving as the primary source [35]. Mendelian randomization studies have established causal relationships between specific IL-17 subtypes and IBD development [36]. Additionally, IL17RA promoter polymorphisms directly correlate with increased cardiovascular mortality, demonstrating the significance of IL-17-mediated immune activation [37]. The cardiac consequences of sustained IL-17 elevation are multifaceted. Experimental models reveal that IL-17 directly impairs myocardial contractility through disrupted calcium handling and activates NF-κB-mediated pathways that drive pathological cardiac remodeling [38]. In ischemic contexts, IL-17 creates a proarrhythmic setting by impairing electrical conduction, prolonging repolarization, and promoting both myocardial fibrosis and inflammation—effects that are reversible with IL-17 neutralization [39]. Additionally, IL-17A exacerbates myocardial ischemia/reperfusion injury through dual mechanisms of enhanced cardiomyocyte apoptosis and neutrophil-mediated tissue damage [40]. Collectively, these findings establish IL-17 as a biomarker increased in IBD and a direct mediator of the cardiac complications that contribute to HF in this patient population.

### 3.7. Interleukin-33

IL-33 is produced by various cells, including epithelial cells, antigen-presenting cells (APCs), and fibroblasts [41,42]. IL-33 influences the immune system, including Th2 cell induction and Th1 cell activation, as well as the modulation of group 2 innate lymphoid cells and regulatory T cells [41]. IL-33 binds to its receptor, the growth stimulus-expressed gene 2 protein (ST2), which produces downstream cascades that can phosphorylate extracellular signal-regulated kinase 1/2, p38 MAPK, and JNKs and activate NF-κB [43]. IL-33 is active in IBD. Its effect on the heart is complex and not fully understood. Interestingly, although IL-33 activates NF-κB, it decreases the NF-κB activation caused by angiotensin II or phenylephrine in cardiomyocytes [43]. Experimental models have demonstrated that IL-33 knockout causes pathologic cardiac remodeling in mice with impaired cardiac function [44]. IL-33 may produce myocardial damage and increase fibrosis. Soluble ST2 (sST2) has been shown to decrease the beneficial effects of IL-33 [45]. In IBD patients, increased sST2 is secreted by intestinal proinflammatory T cells [46], and IBD patients have been found to have decreased serum levels of IL-33 [47]. Put together, the dysregulation of the IL-33/ST2L/sST2 pathway may serve as a shared link between IBD and HF pathogenesis.

## 4. Experimental Evidence

A recent murine model provides mechanistic evidence linking IBD to HF [48]. Two mouse models were used. In the first, chronic colitis was induced in mice by administering 2% dextran sodium sulfate (DSS). In another model, interleukin 10 (IL-10) knockout mice on a 129/Sv background developed spontaneous colitis. Chronic intestinal inflammation reproducibly precipitated a similar cardiac phenotype in both models. Transthoracic echocardiography revealed a significant decrease in left ventricular ejection fraction and fractional shortening, accompanied by inflammatory cell infiltration, myofibrillar disorganization, interstitial edema, and increased cardiac fibrosis [48]. Molecular profiling corroborated a profibrotic switch with an increase in collagen fiber deposition and increased fibrotic proteins, suggesting that chronic colitis promotes cardiac fibrosis [48]. Cardiac fibrosis is an important mechanism leading to HFpEF [49]. Therapeutic fecal microbiota transplantation (FMT) is the process of delivering a gut microbiome from a healthy donor to a recipient in order to restore the gut microbiome in the recipient and manage various disease processes [50]. Studies have demonstrated that FMT from a healthy donor mouse attenuated both gut and cardiac pathology. FMT dampened colonic IL-1β expression and myeloperoxidase activity, improved ventricular systolic indices, and reduced myocardial collagen burden. The reversal in symptoms was near-complete in DSS mice and partial in the IL-10 double knockout model, highlighting a cardioprotective role for endogenous IL-10 [48].

### Intestinal Barrier Dysfunction and Gut Microbiome Alteration

**Figure 3:**
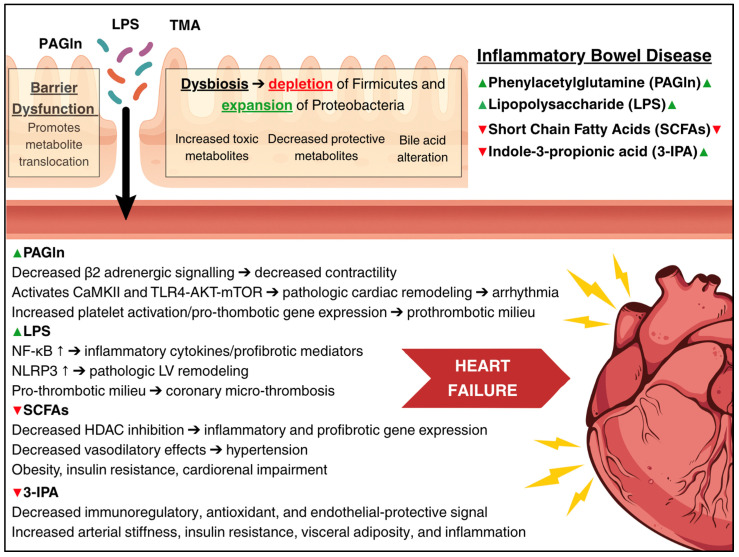
Summary of the effects of intestinal barrier dysfunction and dysbiosis in inflammatory bowel disease on the heart. Arrows (→) indicate mechanistic pathways linking microbial metabolites to cardiac dysfunction. Upward arrows (↑) denote increased cytokine levels in inflammatory bowel disease. Green (▲) and red (▼) arrows represent increased or decreased levels of key metabolites. Dysbiosis, characterized by reduced Firmicutes and increased Proteobacteria, leads to toxic metabolite accumulation and loss of protective metabolites. These changes promote inflammation, fibrosis, and thrombosis, contributing to heart failure.

The gut–heart axis represents a pathway connecting intestinal and cardiac health [51,52]. Two primary mechanisms appear particularly important: (1) intestinal barrier dysfunction, often termed “leaky gut,” and (2) gut microbiome dysbiosis. Together, these alterations result in increased translocation of metabolites into the systemic circulation, enhanced production of toxic metabolites, reduced production of protective metabolites, and disruption of metabolite homeostasis. Figure 3 summarizes the key metabolites that are altered in IBD and have established roles in HF pathogenesis.

## 5. Intestinal Barrier Dysfunction

### 5.1. Structural and Functional Changes

The intestinal barrier comprises a multilayered selectively permeable system including protective mucus, epithelial cells joined by tight junctions, antimicrobial molecules, and mucosal immune networks [53]. IBD is characterized by intestinal inflammation, leading to barrier dysfunction that may clinically manifest as diarrhea and impaired absorptive capacity that can result in nutritional deficiencies [54]. In IBD, several factors contribute to the disruption of the intestinal barrier. These include alterations to tight junctions [55], increased epithelial cell apoptosis, erosions, and mucosal abnormalities [54,56]. Increased enteric permeability has been described in healthy first-degree relatives of patients with CD, supporting a genetic basis for barrier dysfunction [57]. Additionally, intestinal barrier dysfunction appears to scale with inflammatory activity in IBD [58,59]. Increased intestinal permeability results in translocation of luminal pathogens and agents into systemic circulation, triggering an inflammatory response [60]. The translocation of toxic microbial products and associated immune activation contributes to the development of HF.

### 5.2. Cardiovascular Implications

Intestinal barrier dysfunction has been observed in cardiovascular disease (CVD) states. A recent meta-analysis demonstrated that patients with CVD have significantly higher circulating levels of lipopolysaccharide (LPS), d-lactate, zonulin, and serum diamine oxidase, markers of intestinal permeability, relative to healthy controls [61]. This suggests that a breached intestinal barrier, whether caused by gut inflammation or by cardiac disease, correlates with systemic inflammation. In chronic HF, intestinal wall edema from venous congestion can itself induce intestinal barrier dysfunction, exacerbating systemic inflammation via microbial translocation [62]. Intestinal barrier dysfunction in IBD allows luminal contents to bypass epithelial tight junctions and evade mucosal immune surveillance, leading to increased systemic exposure to microbial products and endotoxins. This promotes endotoxemia, drives systemic inflammation, and facilitates translocation of toxins to distant organs, including the heart [63].

### 5.3. Lipopolysaccharide and Cardiac Dysfunction

LPS, an endotoxin from Gram-negative bacterial outer membranes, represents a key mediator linking barrier dysfunction in IBD to HF [64,65]. Circulating LPS binds Toll-like receptor-4 on endothelial cells and cardiomyocytes, triggering NF-κB–driven proinflammatory cytokine release, profibrotic signaling, a pro-thrombotic milieu, and mitochondrial dysfunction. Animal models demonstrate that LPS exposure promotes myocardial fibrosis and HFpEF through multiple mechanisms, including increased TNF-α and IL-6 [66]. Inhibition of this pathway in an LPS-treated mouse model led to cardioprotective outcomes [67]. In rodent and in vitro models, LPS exposure amplified components of the NLRP3 inflammasome and increased NLRP3 gene expression, respectively [68]. Activation of the NLRP3 inflammasome in these models produced HFpEF-like phenotypes characterized by concentric remodeling, LV stiffening, and impaired relaxation [68]. Additionally, LPS creates a pro-thrombotic environment, primarily through contact pathway and platelet activation [69,70,71]. This promotes coronary microvascular thrombosis [72], accelerating adverse ventricular remodeling and HF progression. In a sepsis model, LPS led to cardiac dysfunction through mitochondrial electron transport chain disruption, increased ROS generation, and impaired antioxidant defenses, resulting in oxidative damage and lipid peroxidation [73]. Clinically, LPS levels increase significantly during decompensated HF episodes, likely due to splanchnic venous congestion, and decrease with successful decongestion [74,75]. This temporal relationship suggests that LPS may serve both as a biomarker and mediator of HF progression.

### 5.4. Does TMAO Play a Role?

Intestinal barrier dysfunction in IBD also allows for translocation of other gut-derived metabolites with well-established roles in HF pathophysiology. Some authors have speculated that in the setting of IBD-related gut barrier dysfunction, increased trimethylamine (TMA) absorption, and thus trimethylamine N-oxide (TMAO) production, may contribute to increased HF risk [76]. Increased serum TMAO is associated with an increased risk of all-cause mortality in HF [77] and has been associated with increased HF incidence [78]. TMAO promotes cardiac hypertrophy and fibrosis by activating profibrotic signaling pathways, including TGF-β/Smad3 [79] and JAK2-STAT3 [80], and by stimulating the NLRP3 inflammasome in cardiac fibroblasts [81]. TMAO also impairs myocardial energetics [82] and induces oxidative stress and endothelial dysfunction [83]. Interestingly, IBD has been shown to be accompanied by decreased circulating TMAO relative to healthy controls [84]. Additionally, plasma TMAO further decreases in active UC relative to inactive disease [84,85]. This finding of “low TMAO despite leaky gut” may be explained by two converging mechanisms: (1) dysbiosis-driven depletion of choline- and carnitine-metabolizing microbes that encode the cutC and cntA genes, limiting luminal production of trimethylamine (TMA) [84,86]; (2) systemic inflammation suppresses hepatic expression of flavin-containing monooxygenase 3 (FMO3), the key enzyme responsible for converting TMA to TMAO [87]. In the dextran sulfate sodium (DSS) colitis model, colonic tissue TMA levels increased while TMAO concentrations declined, a pattern mediated by downregulation of hepatic FMO3 expression—highlighting an inflammation-induced impairment of TMAO synthesis during active IBD [88]. As a result, although barrier dysfunction enhances the translocation of many gut-derived molecules, the systemic TMAO pool contracts in active IBD, distinguishing it from other forms of gut-derived metabolic injury. Despite lower circulating TMAO levels in IBD, recent evidence suggests that even elevations within this suppressed range may still contribute to vascular dysfunction [89]. In particular, higher TMAO levels were associated with impaired coronary microvascular function in patients with UC [89], highlighting a potential role for TMAO in IBD-related cardiovascular risk even when absolute concentrations are reduced. Interestingly, a metabolomics study of Italian UC patients demonstrated increased fecal TMAO [90], although it is unclear if fecal TMAO correlates with plasma TMAO. Further research is needed to clarify TMAO’s role at the intersection of IBD and HF pathogenesis.

## 6. Gut Microbiome Dysbiosis

### 6.1. Microbial Composition Changes

IBD is characterized by significant dysbiosis, with depletion of Firmicutes (including butyrate-producing genera like Faecalibacterium and Roseburia) and expansion of Proteobacteria such as Enterobacteriaceae [91,92]. This disrupts the metabolic output of the gut microbiome, promoting downstream cardiovascular consequences.

### 6.2. Short-Chain Fatty Acid Depletion

Short-chain fatty acids (SCFAs) such as acetate, propionate, and butyrate are beneficial microbial metabolites produced by fermentation of dietary fiber [93]. SCFAs function as primary energy substrates for colonocytes, enhance epithelial barrier integrity, and regulate both innate and adaptive immune responses to mitigate excessive inflammation [93]. Following absorption, SCFAs participate in hepatic and systemic metabolic processes, modulating gluconeogenesis, lipogenesis, and appetite regulation to maintain glucose and lipid homeostasis [94]. At elevated concentrations, particularly in the colonic microenvironment, SCFAs exert antineoplastic effects [94]. Additionally, SCFAs engage in cellular signaling through multiple pathways, including activation of G-protein-coupled receptors (GPCRs), inhibition of histone deacetylases (HDAC), and stimulation of peroxisome proliferator-activated receptor gamma (PPARγ). Through these mechanisms, they serve as critical mediators linking dietary intake, gut microbial composition, gastrointestinal pathology, and systemic physiological responses [94].

IBD-associated dysbiosis is characterized by a loss of SCFA-producing bacteria and significantly reduced SCFA levels in the gut [93]. A recent meta-analysis of 12 studies found that patients with IBD exhibited significantly lower fecal concentrations of acetate, propionate, butyrate, and valerate, alongside elevated lactate, relative to healthy controls [95]. Reduced SCFAs in IBD perpetuates impaired epithelial barrier integrity, diminished mucus production, and dysregulation of immune cell activity, including reduced differentiation of regulatory T cells. These deficits contribute to increased intestinal permeability, heightened proinflammatory cytokine release, and impaired tissue repair, thereby perpetuating chronic gut inflammation and mucosal injury [96].

Beyond the gastrointestinal tract, the consequences of SCFA depletion affect cardiovascular and metabolic function, including insulin resistance, blood pressure control, and HF development or progression [97]. SCFAs exert multiple cardioprotective effects relevant to HF progression. SCFAs, specifically butyrate, promote macrophage mitochondrial function and dampen inflammation [98,99]. In a rat model of myocardial fibrosis, dietary supplementation with butyric acid ameliorated myocardial fibrosis by promoting macrophage polarization from a pro- to anti-inflammatory state and restoring mitochondrial function [99]. Additionally, SCFAs have been shown to have a vasorelaxant or vasodilatory effect in human and animal models [100,101,102]. A study of older adults found that increased serum SCFA concentrations, specifically acetic acid and valeric acid, were inversely associated with systolic blood pressure [103], suggesting that reduced SCFA levels in the context of IBD may increase the risk of hypertensive heart disease. SCFAs also exert cardioprotective effects through HDAC inhibition. HDAC inhibition reverses diastolic dysfunction in various animal models [104,105,106]. HDAC inhibition reduces proinflammatory cytokines [107], reduces profibrotic signaling [108], reverses maladaptive protein acetylation, promotes autophagy-related gene expression, and improves energy metabolism [109]. Together, these effects contribute to improved myocardial structure and function. In a diabetic mouse model, sodium butyrate treatment led to significant improvements in ejection fraction, fractional shortening, and reduced myocardial fibrosis and cardiomyocyte hypertrophy [110]. This suggests that the low SCFA levels observed in IBD may impair endogenous HDAC inhibition, linking gut dysbiosis in IBD to adverse cardiac remodeling. Animal models have shown that SCFA supplementation reduces body weight [111,112,113], stimulates insulin sensitivity, reduces hepatic steatosis [112], improves mitochondrial biogenesis, and inhibits chronic inflammation [111]. Dietary interventions resulting in low fecal SCFA levels have been shown to promote adiposity in mice [114]. SCFA depletion may promote obesity by disrupting adipokine signaling and metabolic homeostasis [98]. Obesity is a well-established risk factor for both systolic and diastolic dysfunction, with a particularly strong association with HFpEF [115,116]. Reduced levels of acetate, propionate, and butyrate in HF is associated with lower left ventricular ejection fraction, elevated NT-proBNP, and a reduced glomerular filtration rate, linking SCFA deficiency to cardiac dysfunction, ventricular stress, and cardiorenal impairment [117]. Taken collectively, these mechanisms establish a mechanistic link between microbiome-driven SCFA depletion in IBD and the pathogenesis of HF.

### 6.3. Do Bile Acids Play a Role?

Bile acids play a multifaceted role in the pathogenesis and clinical course of IBD. In IBD, gut microbial imbalance interferes with the normal microbial conversion of bile acids secreted by hepatocytes. As a result, primary bile acids accumulate while levels of secondary bile acids, which exert anti-inflammatory effects, are markedly reduced [118]. The abnormal bile acid profile feeds back to further disturb the gut microbiome, contributing to further dysbiosis and inflammation [118]. These alterations impair the integrity of the intestinal barrier and disrupt immune regulation through impaired signaling of bile acid-activated receptors. Bile acid-activated receptors, particularly the nuclear receptor farnesoid X receptor (FXR) and the G protein-coupled receptor TGR5, are also expressed in cardiomyocytes, endothelial cells, and vascular smooth muscle cells [119]. Dysfunctional FXR and TGR5 signaling pathways can adversely impact cardiac health by promoting atherosclerosis, systemic inflammation, and myocardial metabolic disturbances, which collectively contribute to cardiac dysfunction [120,121,122]. Conversely, in chronic HF, congestion-related intestinal hypoperfusion and hepatobiliary dysfunction disrupt enterohepatic bile acid circulation, leading to a relative increase in circulating secondary bile acids and a reduction in primary bile acids [123]. An elevated secondary-to-primary bile acid ratio has been associated with adverse clinical outcomes, including increased mortality [123]. While IBD and HF exhibit opposite alterations in bile acid profiles, with primary dominant in IBD and secondary dominant in HF, both conditions converge on disrupted bile acid receptor signaling and downstream inflammatory and fibrotic pathways. The net impact of IBD-related changes in bile acid metabolism on the heart is not fully established, and the precise role of bile acid dysregulation in IBD-related HF remains speculative and warrants further investigation.

### 6.4. Phenylacetylglutamine

**Figure 4:**
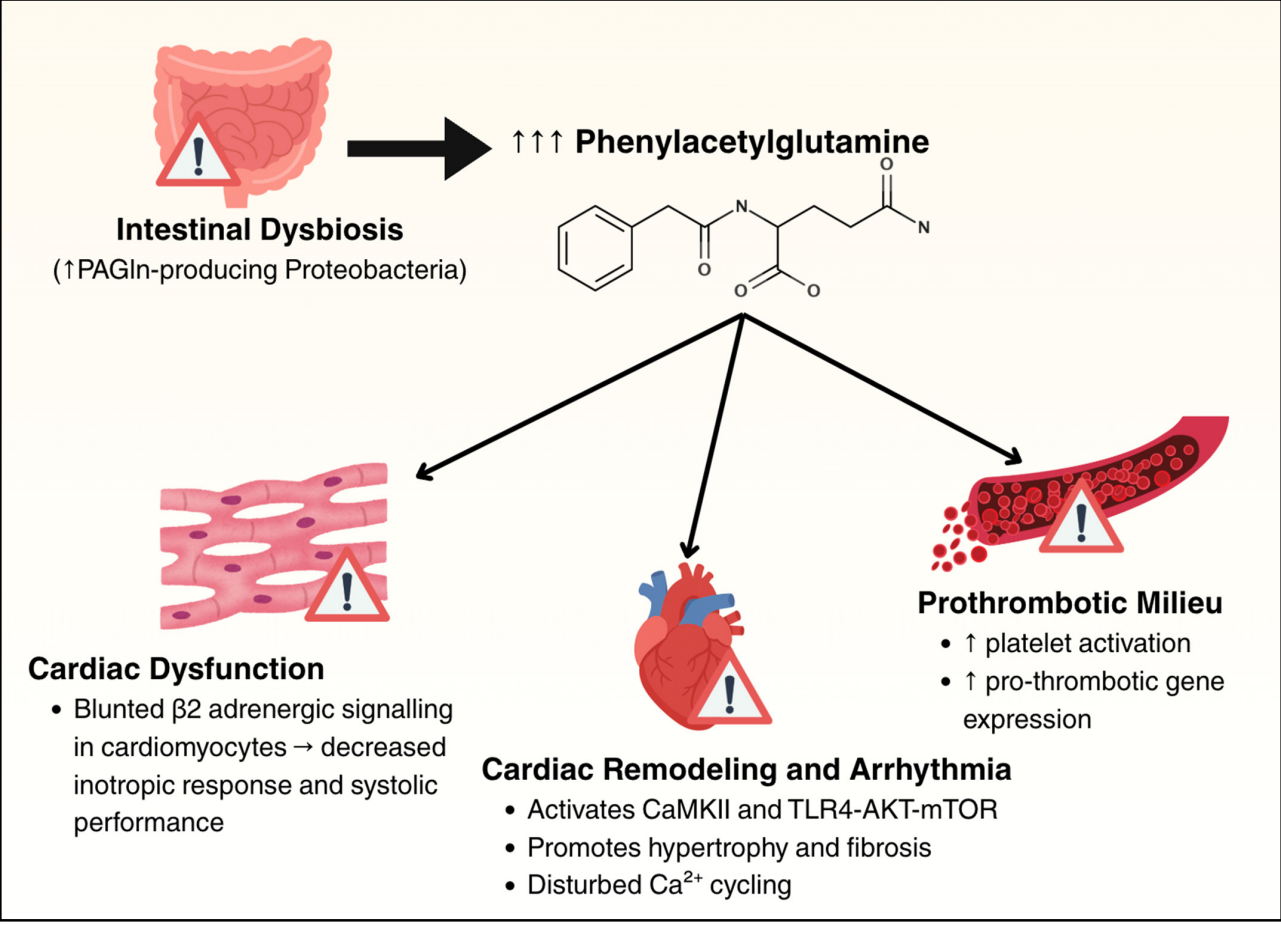
Summary of the effect of elevated phenylacetylglutamine on the heart and thrombotic milieu. Phenylacetylglutamine is produced by Proteobacteria in the setting of dysbiosis, and contributes to cardiac dysfunction, pathologic cardiac remodeling, and prothrombotic milieu. Upward arrows (↑) indicate increased levels or activation of molecular or cellular processes.

PAGln is derived from dietary phenylalanine, which is converted to phenylacetic acid by gut microbes and then conjugated with glutamine in hepatocytes and renal cells. PAGln levels are elevated in patients with CD relative to healthy controls [124]. In treatment-naïve CD, intestinal dysbiosis leads to the expansion of PAGln-producing Proteobacteria, resulting in elevated circulating PAGln levels [124]. Experimental models using the murine analog phenylacetylglycine (PAGly) demonstrate that exogenous administration exacerbates colitis severity, while human ex vivo studies show that PAGln promotes platelet activation and enhances pro-thrombotic gene expression [124]. Beyond the gastrointestinal tract, PAGln interacts with host adrenergic signaling pathways; it functions as a negative allosteric modulator of β2-adrenergic receptors in murine and human ventricular cardiomyocytes, attenuating cardiac contractility [125]. Elevated plasma PAGln levels have been independently associated with increased risk of hospitalization and mortality in patients with HF [126]. Mechanistically, PAGln has been shown to worsen adverse cardiac remodeling and increase susceptibility to arrhythmias via activation of CaMKII and TLR4-AKT-mTOR signaling pathways in murine models of HF [127,128]. In patients with HF circulating PAGln levels correlate with the degree of systolic dysfunction, with the highest concentrations observed in patients with HFrEF, followed by those with mildly reduced EF. PAGln levels are elevated in HFpEF as well, though to a lesser extent [129]. This gradient supports a potential role for PAGln in the progression of myocardial dysfunction, particularly in the context of impaired contractility. These findings suggest that increased PAGln levels in the setting of IBD represent a link between gut inflammation and heightened HF risk. Figure 4 summarizes the effects of PAGln on the heart and vasculature.

### 6.5. Tryptophan-Derived Indoles

Dysbiosis in IBD also perturbs bacterial tryptophan metabolism, reducing levels of beneficial indole derivatives. Indole-3-propionic acid (IPA), a microbiota-produced indole, is significantly decreased in patients with active IBD compared to healthy controls [130]. High IPA levels are negatively associated with arterial stiffness, insulin resistance, fasting glucose, and visceral fat [131]. Its deficiency removes an important immunoregulatory signal, potentially exacerbating systemic inflammation [132]. Consequently, chronic depletion of IPA due to persistent intestinal dysbiosis in IBD could promote systemic metabolic dysfunction, heightened inflammatory signaling, adverse myocardial remodeling, and, ultimately, increased risk of HF.

### 6.6. Summary of Dysbiosis and Barrier Dysfunction

IBD is characterized by gut dysbiosis and gut barrier dysfunction, both of which are associated with increased cardiovascular event rates, including HF, and coronary artery and cerebrovascular disease [63]. Experimental models have shown that modulating the microbiome (with antibiotics, probiotics, or FMT) can influence cardiac outcomes [48]. However, the specific microbial metabolites and mechanistic pathways driving these effects in humans remain incompletely defined. The hypothesis that restoring a single microbial genus in humans with IBD could reduce HF risk remains speculative. What is more robustly supported is the interplay between dysbiosis and intestinal barrier dysfunction [63]. A dysbiotic microbiota can cause barrier dysfunction, and, in turn, a leaky barrier increases systemic exposure to dysbiotic microbial products. This bidirectional relationship perpetuates chronic inflammation, likely amplifying systemic inflammatory load, contributing to HF pathogenesis.

## 7. Endothelial and Vascular Dysfunction and Thrombotic Risk

Chronic inflammation in IBD affects endothelial cell function and microcirculation, creating conditions conducive to HF development through both direct myocardial effects and accelerated atherosclerosis.

### 7.1. Systemic Endothelial Dysfunction

Chronic inflammation in IBD adversely affects the endothelium and microcirculation, which are key contributors to HF pathogenesis. Persistent elevation of inflammatory cytokines impairs endothelial function by promoting leukocyte adhesion, increasing oxidative stress, and reducing NO bioavailability [133,134]. At the cellular level, chronic inflammation activates endothelial cells, increasing the expression of adhesion molecules such as ICAM-1 and VCAM-1, which facilitate leukocyte adhesion and transmigration [135,136]. Infiltrating leukocytes release cytokines and reactive oxygen species, disrupting endothelial nitric oxide synthase function and reducing NO availability [135,136]. This promotes oxidative stress, increases permeability, and shifts the endothelium toward a pro-thrombotic, vasoconstrictive state [135,136], key features of endothelial dysfunction contributing to HF pathogenesis. These mechanisms are clinically relevant, as patients with IBD consistently demonstrate impaired endothelial function even in the absence of traditional cardiovascular risk factors [137]. Flow-mediated dilation of the brachial artery—a validated marker of endothelial health—is significantly reduced in IBD patients, while pulse wave velocity is increased, indicating early vascular dysfunction and heightened atherosclerotic risk [137].

### 7.2. Coronary Microvascular Dysfunction

Endothelial dysfunction extends to the coronary microvasculature. Impaired coronary microvascular function has been observed in IBD, with reduced coronary flow reserve reported in one study [138]. This can contribute to myocardial ischemia in the absence of overt coronary artery disease or non-obstructive coronary artery disease (NOCAD), a phenomenon observed in a number of patients with HFpEF [139].

### 7.3. Accelerated Atherosclerosis and Thrombotic Risk

IBD functions as an independent risk factor for atherosclerotic cardiovascular disease (ASCVD) and thrombotic disease [140,141,142]. This occurs through multiple interrelated mechanisms, including elevated proinflammatory cytokines, upregulation of adhesion molecules, promotion of leukocyte adhesion and vascular inflammation, and gut microbiome and barrier dysfunction [140]. The proinflammatory milieu creates a hypercoagulable state through abnormal platelet function, impaired fibrinolysis, and activation of the coagulation cascade, increasing the risk of arterial and venous thrombosis [140]. This is further exacerbated by corticosteroid use, commonly used in IBD treatment [140]. Together, these processes accelerate atherogenesis and increase the risk of ischemic HF.

## 8. Hemodynamic and Autonomic Perturbations

IBD patients exhibit alterations in hemodynamic status and autonomic function that may contribute to cardiac dysfunction through distinct but complementary mechanisms.

### 8.1. Hemodynamic Changes

Chronic anemia, common in IBD due to gastrointestinal blood loss and chronic inflammation, reduces blood oxygen-carrying capacity and triggers compensatory cardiovascular responses, including tachycardia and increased cardiac output [143]. While initially adaptive, sustained high-output states can impose chronic volume stress, leading to ventricular dilatation and eventual systolic dysfunction [143].

### 8.2. Autonomic Nervous System Dysfunction

In IBD, autonomic dysfunction is evidenced by a significant reduction in heart rate variability (HRV) compared to healthy controls [144]. This is in part due to parasympathetic suppression, which leads to sympathetic predominance [145]. This dysfunction appears bidirectionally linked to TNF-α levels [146]. The vagus nerve releases acetylcholine, which normally exerts anti-inflammatory effects through alpha 7 nicotinic receptors (α7nAchRs) on human macrophages that inhibit the production of NF-κB and cytokines, including TNF-α [147,148]. Reduced vagal activity impairs this inhibition, resulting in increased TNF-α production. Conversely, TNF-α can modulate vagal neurocircuitry in the brainstem, affecting autonomic function and potentially contributing to autonomic dysregulation [149,150]. Over time, chronic sympathetic hyperactivity may contribute to adverse cardiac remodeling [151]. In chronic HF, prolonged sympathetic activation leads to persistent stimulation of β1-adrenergic receptors, causing desensitization and downregulation via upregulation of GRK2, reducing cardiac contractility [152]. GRK2 also impairs α2-adrenergic receptor function, removing inhibitory feedback on norepinephrine release, further increasing sympathetic drive [152]. Meanwhile, the protective β2-receptor signaling becomes disrupted, and adrenal glands over-secrete catecholamines due to α2-adrenergic receptor dysfunction. This sustained catecholamine exposure results in calcium overload, oxidative stress, and cardiomyocyte apoptosis, accelerating HF progression [152]. However, studies examining the role of ANS dysfunction in IBD-related cardiac dysfunction specifically are currently lacking.

## 9. Nutritional Sequelae

**Figure 5:**
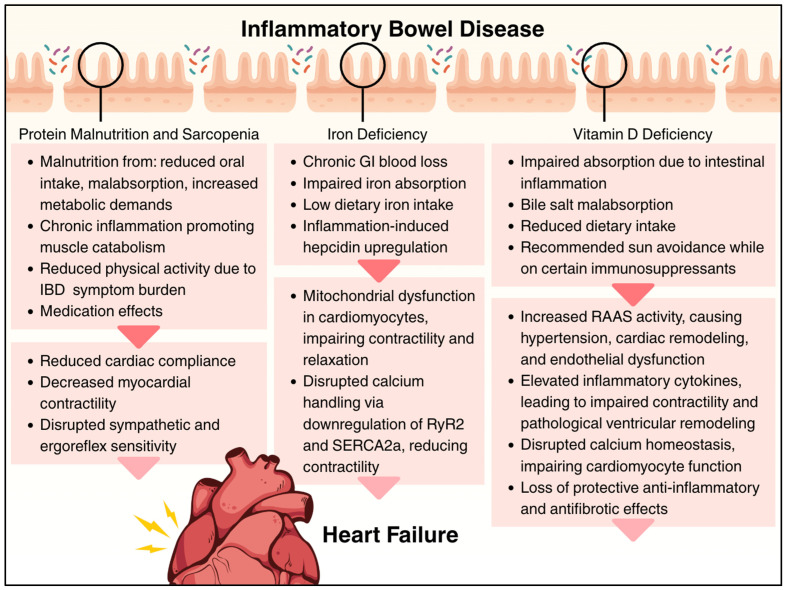
Summary of the nutritional sequelae of inflammatory bowel disease and how they contribute to cardiac dysfunction. Circles highlight that intestinal pathology contributes to malabsorption and nutrient deficiency, including protein malnutrition, sarcopenia, iron deficiency, and vitamin D deficiency. Downward arrows (▼) indicate stepwise progression from nutritional deficiencies to cardiac effects and ultimately heart failure.

Chronic intestinal inflammation in IBD is associated with nutritional disturbances that have direct relevance to cardiovascular health. Even in the absence of active gastrointestinal symptoms, patients with IBD frequently exhibit evidence of protein–energy malnutrition, sarcopenia, and micronutrient deficiencies [153,154,155,156]. The cumulative burden of IBD-related nutritional disturbance creates an environment that is permissive to the development or progression of HF, which highlights the importance of early nutritional intervention as a potential strategy for HF prevention in this high-risk population. Figure 5 summarizes the nutritional sequelae of IBD and how they contribute to increased HF risk.

### 9.1. Protein Malnutrition and Sarcopenia

Protein–energy malnutrition is common in IBD, with one systematic review estimating the prevalence of myopenia and sarcopenia at 42% and 17%, respectively [157]. Patients with IBD are at increased risk for sarcopenia due to a multifactorial interplay of mechanisms, including reduced physical activity due to symptom burden, medication effects, a proinflammatory state, which promotes muscle catabolism, and malnutrition resulting from reduced oral intake, malabsorption, and increased metabolic demands [158]. In a canine model, protein–calorie malnutrition impaired left ventricular function by decreasing compliance due to starvation-induced edema and reduced contractility from myofibrillar atrophy [159]. Sarcopenia can contribute to HF through altered sympathetic and ergoreflex sensitivity, disrupting the regulation of ventilation, cardiovascular responses during physical activity, and metabolic dysregulation [160,161]. Interestingly, proinflammatory factors that are elevated in IBD and HF, including TNF-α, IL-6, and IL-1, can accelerate muscle atrophy through the autophagy–lysosome and ubiquitin–proteasome systems, ultimately contributing to the development of sarcopenia [161]. In established HF, sarcopenia is also associated with a 64% increase in poor prognosis, including mortality and hospitalization [162]. Most research on the link between protein malnutrition and HF development is limited to studies in children with severe malnutrition, particularly kwashiorkor, which has been associated with mild dilated cardiomyopathy [163]. Additionally, among individuals hospitalized with HF, protein–energy malnutrition frequently co-occurs and contributes to significantly worse health outcomes [164].

### 9.2. Micronutrient Deficiencies

Several micronutrient deficiencies commonly observed in IBD have known deleterious effects on cardiovascular function and contribute to comorbid progression of established HF. IBD-related iron deficiency and iron deficiency anemia result from chronic gastrointestinal blood loss, impaired absorption, inadequate dietary intake, and inflammation-induced hepcidin upregulation [165]. Iron deficiency in HF is independently associated with worse symptoms, reduced exercise capacity, poorer quality of life, increased risk of hospitalization, and higher mortality, regardless of the presence of anemia [166,167,168]. Iron deficiency directly affects cardiomyocytes through mitochondrial dysfunction, impairing both contractility and relaxation [169]. Iron deficiency also alters calcium handling in cardiomyocytes by downregulating RyR2 and SERCA2a, further weakening contractility [170].

Vitamin D deficiency is disproportionately prevalent in IBD due to several factors, such as impaired nutrient absorption from intestinal inflammation, bile salt malabsorption, and lack of sunlight exposure [171,172]. Vitamin D exerts anti-inflammatory and antifibrotic effects [173] and plays a role in regulating glycemic control and enhancing insulin sensitivity [174]. Vitamin D deficiency promotes increased renin–angiotensin–aldosterone system activity, which can contribute to HF through increased hypertension, cardiac remodeling, and endothelial dysfunction [175]. Vitamin D deficiency also leads to increased proinflammatory cytokines, including IL-6 and TNF-a [176], leading to impaired contractility [24] and pathologic ventricular remodeling [16]. Lastly, vitamin deficiency disrupts intracellular calcium homeostasis, which can subsequently affect the function of cardiomyocytes, as well as alters PTH levels, causing myocardial fibrosis and hypertrophy [177].

Additionally, rare cases of micronutrient-related reversible cardiac dysfunction have been reported in IBD, particularly in patients requiring parenteral nutrition. Documented complications include selenium deficiency-induced cardiomyopathy [178] and thiamine deficiency-associated wet beriberi [179]. While uncommon, these cases underscore the potential severity of nutrition-related cardiac complications in IBD.

## 10. Shared Genetic and Epigenetic Susceptibility

### 10.1. Shared Genetic Loci

IBD and HF share genetic susceptibility loci that influence overlapping biological pathways. Nucleotide-binding oligomerization domain-containing protein 2 (NOD2) is a cytosolic pattern recognition receptor that plays a central role in the pathogenesis of IBD [180,181]. NOD2 is expressed in monocytes, macrophages, and dendritic cells involved in immune responses, as well as in certain epithelial cells, such as those of the intestinal lining. NOD2 recognizes muramyl dipeptide, a conserved motif in bacterial peptidoglycan, and upon activation, modulates immune responses in the gut. Loss-of-function mutations in NOD2 are strong genetic risk factors for CD [180,181]. NOD2 also modulates cardiovascular inflammation. NOD2-knockout mice develop significantly worsened cardiac hypertrophy and fibrosis under pressure overload, suggesting NOD2 signaling normally plays a protective role in attenuating cardiac remodeling by modulating multiple inflammatory and fibrotic signaling cascades [182]. A recent Mendelian randomization study provided genetic evidence supporting a causal relationship between UC and HF. Using four independent GWAS datasets of European ancestry, the study demonstrated a consistent association between genetically predicted UC and increased HF risk. In contrast, reverse MR analyses did not support a causal effect of HF on UC. Together, these findings suggest a unidirectional genetic predisposition linking UC to elevated HF risk [183]. Additionally, several susceptibility loci implicated in IBD converge on immune signaling pathways that are also relevant in cardiovascular pathology. One such pathway is the IL-23/Th17 axis. Polymorphisms in IL23R and downstream effectors such as TYK2 and STAT3 confer protection against IBD by attenuating Th17-mediated immune activation [184]. Conversely, hyperactivation of the IL-23/IL-17 pathway contributes to persistent intestinal inflammation and has been linked to adverse cardiac remodeling and myocardial fibrosis, which can lead to HF [38,39,40,185].

### 10.2. MicroRNA Dysregulation

A novel mechanism linking IBD and HF involves microRNA dysregulation in cardiac tissue. miRNAs are small noncoding RNAs that regulate gene expression [186]. Chronic intestinal inflammation leads to dysregulation of numerous miRNAs in both local tissues and circulation, which can lead to cardiac dysfunction [76]. Experimental rat models of dextran sodium sulfate (DSS)-induced colitis have revealed that chronic colitis leads to significant upregulation of 56 microRNAs in the heart [9]. A subset of these miRNAs suppressed myocardial and serum brain-derived neurotrophic factor (BDNF) [9]. BDNF is markedly decreased in HF, which is associated with increased NT-proBNP, adverse cardiac remodeling, and disease progression [187]. miR-21 is upregulated in IBD-affected colonic tissues, which promotes proinflammatory pathways [188]. In the myocardium, miR-21 is known to activate cardiac fibroblasts and enhance collagen deposition and may play a role in maladaptive remodeling, causing HF [189,190]. miR-155 is upregulated in the inflamed colonic tissue and peripheral immune cells of patients with active IBD [191]. miR-155 expression in macrophages contributes to cardiac pathology via myocardial inflammation, fibrosis, and adverse remodeling and HF [192,193]. Overall, miRNA dysregulation provides a molecular link between IBD and myocardial remodeling. IBD triggers an array of miRNAs that not only contribute to intestinal inflammation but also systemically impact the heart’s gene expression profile.

### 10.3. Pharmacology

The relationship between IBD therapeutics and cardiovascular outcomes represents a critical clinical consideration, with both protective and harmful effects documented across different classes of medications used in IBD management. The most important principle is that active IBD inflammation dramatically increases HF risk, with disease flares associated with a 2.5-fold increase in incident HF [6]. Increased inflammation, marked by elevated inflammatory markers such as C-reactive protein, IL-6, and TNF-α, has been independently associated with HF incidence [194]. This emphasizes that achieving and maintaining disease remission should be considered a cardiovascular protective strategy. In IBD, exposure to systemic corticosteroids was associated with a markedly increased risk of both acute myocardial infarction and HF, suggesting that corticosteroid use, potentially a surrogate for disease severity, may contribute to cardiovascular risk [4]. Systemic corticosteroids are the cornerstone of acute IBD flare management but carry well-established cardiometabolic consequences. These include hyperglycemia, hypertension, weight gain, dyslipidemia, metabolic syndrome, and increased risk of cardiovascular events including HF [195,196]. Given this, minimizing cumulative steroid exposure through early initiation of steroid-sparing therapies is essential for long-term cardiovascular protection. In general, steroid-sparing agents may reduce cardiovascular risk by controlling inflammation, although certain agents require caution in specific populations, particularly those with established cardiovascular disease or risk factors. Of particular interest are anti-TNF therapies, which provide effective control of systemic inflammation and may confer cardiovascular benefit. In a large French cohort, anti-TNF use was associated with a reduction in first-time acute arterial events [197]. However, certain trials have described decompensated HF after anti-TNF initiation, particularly in patients with pre-existing left ventricular dysfunction; thus some societies recommend avoiding anti-TNF therapy in patients with established moderate-to-severe HF [198].

### 10.4. Clinical Implications

IBD is a systemic disorder that carries several cardiovascular implications, including HF risk. HF risk is particularly elevated during active disease flares, periods of prolonged corticosteroid exposure, and in the presence of traditional cardiovascular risk factors. Currently, routine cardiovascular screening has not been recommended in IBD-specific guidelines. Instead, risk factor assessment and management are individualized based on patient comorbidities. However, periods of active disease require increased monitoring for the development of HF. Maintaining IBD remission is the most effective HF prevention strategy. Traditional HF prevention strategies, including blood pressure control, diabetes management, lipid optimization, and smoking cessation, remain significant and should be integrated with IBD-specific care. Integrated management involving gastroenterologists, cardiologists, and primary care is vital.

## 11. Future Directions

Gaps remain in understanding the pathogenesis of HF in IBD. Longitudinal cohort studies are needed to further evaluate the impact of therapeutic exposures on cardiac structure and function. Further research is needed to assess biomarkers that are associated with HF risk in IBD patients and their utility in risk stratification. Further metagenomics and metabolomic studies can provide further details of the gut–heart axis in IBD and help determine potential molecular targets. Future research should explore and evaluate the role of targeted interventions in modulating HF risk in IBD. Exploring the effect of targeted interventions, including modulation of systemic inflammation using biologics and microbiome-directed therapies, on cardiac function could help determine their effect on reversing pathological cardiac remodeling. In addition, implementation studies examining the role of integrated care models, such as joint IBD–cardiology clinics, may inform strategies to reduce cardiovascular complications and improve long-term outcomes in this population.

## 12. Conclusions

IBD is increasingly recognized as a systemic disorder with direct implications for HF development, driven by shared inflammatory, metabolic, and genetic pathways. Key cytokines and gut-derived factors activate signaling cascades in the heart, promoting oxidative stress, cardiomyocyte dysfunction, and fibrotic remodeling. Nutritional deficits, vascular dysfunction, and autonomic dysfunction further exacerbate cardiac vulnerability. These interconnected mechanisms can contribute to both HFpEF and HFrEF phenotypes. Given the interrelationship between IBD and HF, both cardiovascular surveillance and early intervention in established IBD are essential. Effective HF prevention in IBD requires integrated care that addresses not only traditional cardiovascular risks but also considers the role of the gut–heart axis. Integrating these insights, along with HF risk stratification and prevention, into IBD care offers the potential to reduce the burden of HF in this vulnerable population.

## Data Availability

No new data were created or analyzed in this study.
